# CLIC2α Chloride Channel Orchestrates Immunomodulation of Hemocyte Phagocytosis and Bactericidal Activity in *Crassostrea gigas*

**DOI:** 10.1016/j.isci.2020.101328

**Published:** 2020-06-30

**Authors:** Xiangyu Zhang, Fan Mao, Nai-Kei Wong, Yongbo Bao, Yue Lin, Kunna Liu, Jun Li, Zhiming Xiang, Haitao Ma, Shu Xiao, Yang Zhang, Ziniu Yu

**Affiliations:** 1 CAS Key Laboratory of Tropical Marine Bio-resources and Ecology, Guangdong Provincial Key Laboratory of Applied Marine Biology, Innovation Academy of South China Sea Ecology and Environmental Engineering (ISEE), South China Sea Institute of Oceanology, Chinese Academy of Science, Guangzhou 510301, P. R. China; 2 Southern Marine Science and Engineering Guangdong Laboratory (Guangzhou), Guangzhou 510301, P. R. China; 3 National Clinical Research Center for Infectious Diseases, Shenzhen Third People's Hospital, The Second Hospital Affiliated to Southern University of Science and Technology, Shenzhen 518112, P. R. China; 4 Zhejiang Key Laboratory of Aquatic Germplasm Resources, College of Biological and Environmental Sciences, Zhejiang Wanli University, Ningbo 315100, P. R. China; 5 University of Chinese Academy of Sciences, Beijing 100049, P. R. China

**Keywords:** Cell Biology, Immunology, Microbiology

## Abstract

Chloride ion plays critical roles in modulating immunological interactions. Herein, we demonstrated that the anion channel CLIC2α mediates Cl^−^ flux to regulate hemocytes functions in the Pacific oyster (*Crassostrea gigas*). Specifically, during infection by *Vibrio parahemolyticus*, chloride influx was activated following onset of phagocytosis. Phosphorylation of Akt was stimulated by Cl^−^ ions entering host cells, further contributing to signal transduction regulating internalization of bacteria through the PI3K/Akt signaling pathway. Concomitantly, Cl^−^ entered phagosomes, promoted the acidification and maturation of phagosomes, and contributed to production of HOCl to eradicate engulfed bacteria. Finally, genomic screening reveals CLIC2α as a major Cl^−^ channel gene responsible for regulating Cl^−^ influx in oysters. Knockdown of CLIC2α predictably impeded phagosome acidification and restricted bacterial killing in oysters. In conclusion, our work has established CLIC2α as a prominent regulator of Cl^−^ influx and thus Cl^−^ function in *C. gigas* in bacterial infection contexts.

## Highlights

• Influx of chloride ions is switched on during phagocytosis in oyster hemocytes
• PI3K/Akt signaling pathway mediates chloride-dependent activation of phagocytosis
• Cl^−^ promotes phagosomal acidification and HOCl production
• CLIC2α is the principal chloride channel encoding gene within oyster genome

## Graphical Abstract

**Figure:**
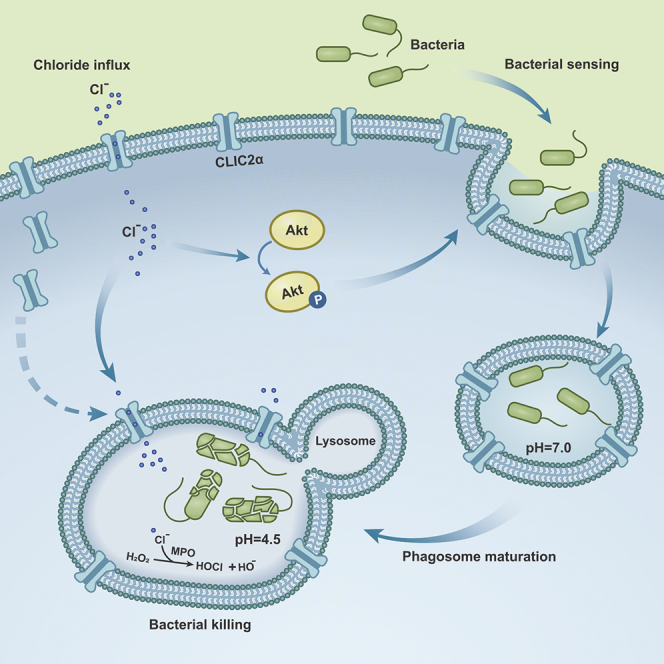

## Introduction

As a principal inorganic anion in the intra- and extracellular environments, chloride (Cl^−^) is involved in an extraordinary range of physiological functions including body fluid retention/excretion, osmotic maintenance, cell volume regulation, and pH balance (Bohn and de Morais, 2017; Rodan, 2019; Wang, 2016). Accumulating evidence has implicated transmembrane Cl^−^ fluxes in antimicrobial processes within immune effectors such as phagocytes, although the underlying mechanisms are not fully understood (Wang, 2016). In mammalian macrophages, phagocytosis is of fundamental importance to innate defenses against invading microbes (Hartenstein and Martinez, 2019). Notably, acidification of phagosomes governs their maturation and eventual antimicrobial capacity (Bouvier et al., 1994), in which Cl^−^ flux is critical to phagosomal pH control and bacterial infection outcomes (Di et al., 2006). Meanwhile, activation of endolysosomal proteases temporally matches the maturation of phagosomes to ensure efficient destruction of engulfed bacteria (Pillay et al., 2002). Some immune-related enzymes, such as cathepsins, show a dependency on Cl^−^ flux for activity via binding to Cl^−^ ion (Cigic and Pain, 1999). In addition, Cl^−^ directly participates in the production of chlorine-containing oxidants for microbial killing in host immunity. Specifically, in neutrophils, myeloperoxidase (MPO), which is enriched in phagosomes, catalytically converts hydrogen peroxide (H_2_O_2_) and Cl^−^ into the highly potent hypochlorous acid (HOCl), which oxidatively decimates microbes via protein chlorination (Busetto et al., 2007; Rosen et al., 2002, 2009).

Owing to its intrinsic properties of a negatively charged ion, Cl^−^ cannot autonomously permeate cellular membranes; its compartmental distribution instead has to depend on passive transport through specific channels or transporters (Stauber et al., 2012). Since the discovery of the first intracellular Cl^−^ channel protein (p64, later renamed as CLIC5B) in bovines, more members of CLIC proteins were subsequently identified in all phyla, wherein six CLIC genes were found in humans (Landry et al., 1989; Redhead et al., 1992). The CLIC family located in various organelles of the cell is involved in physiological functions and pathological conditions in various human diseases, such as tumor onset and progression, Alzheimer's disease, and cardiac dysfunction (Flores-Tellez et al., 2015; Hernandez-Fernaud et al., 2017; Novarino et al., 2004; Peretti et al., 2015; Takano et al., 2012).

Not surprisingly, vertebrate CLICs seem to fulfill indispensable functions as ion channels in helping cells maintain osmotic balance at vesicles and cytoplasm and coordinate interactions between membranes and cytoskeleton (Littler et al., 2010). For example, when low vesicular pH is required, CLICs serve to regulate the actions of proton pumps. CLICs are themselves necessary for the formation and maintenance of intracellular membrane-enclosed vesicles. Elsewhere, substantial evidence based on loss of function of CLIC supports the central importance of these chloride channels in phagocytic defenses. In mice, CLIC1^−/−^ macrophages failed to acidify phagosomes, consequently impairing the cells' capacities for phagosomal proteolysis and reactive oxygen species (ROS) production (Jiang et al., 2012). Likewise, both mice polymorphonuclear leukocytes (PMNs) and human PMNs displayed compromised phagocytic abilities, following CLCN3 knockout (Moreland et al., 2006). In microglial cells, chloride channel blockers suppress formation of engulfment pseudopodia (Harl et al., 2013). Furthermore, CLIC3^−/−^ PMNs also had remarkably reduced NADPH oxidase activity and restricted transendothelial migration. Other than the CLIC family, it should be noted that another specialized Cl^−^ channel, cystic fibrosis transmembrane conductance regulator (CFTR), was initially identified as a regulator of salt transport across epithelial membranes (Jovov et al., 1995). In patients with cystic fibrosis, the reduced expression of CFTR impaired phagocytosis ability in macrophages (Jovov et al., 1995; Pong et al., 2014). In CFTR-null mutants, macrophages failed to lower phagosomal pH or exhibit adequate bactericidal activity. However, loss of function of CFTR did not seem to impact phagocytic abilities or ROS generation in macrophages (Aiken et al., 2012; Deriy et al., 2009; Di et al., 2006), raising the possibility that different chloride channels may have intrinsically distinct molecular mechanisms for governing aspects of phagocytic defenses. This assumption also applies to the CLC-3 gene, which is expressed in neutrophils as plasma membrane chloride channels, with a clear role in supplying neutralizing anion currents for V-type H^+^-ATPases that acidify compartments of endosomal/lysosomal pathways (Scheel et al., 2005). In macrophages, CLIC3 was also found essential for CRIg-mediated *Listeria monocytogenes* (LM) killing by directly interacting with the cytoplasmic domain of CRIg (Kim et al., 2013). Indeed, macrophage-specific rescue and knockdown confirmed that CLIC-deficient macrophages failed to clear bacteria (Monahan and Silverman, 2017).

Chloride flux can govern specific signaling pathways to regulate its physiological functions. For example, activation of the ClC-3 chloride channel, responsible for inducing inhibition of the PI3K/Akt/mTOR signaling pathways, has also been proven to determine apoptosis in human nasopharyngeal carcinoma cell lines (CNE-1, CNE-2Z) (Liu et al., 2013). Over-expression of calcium-activated chloride channel A4 (CLCA4) could inhibit cell migration and invasion by suppressing epithelial-mesenchymal transition (EMT) via the PI3K/ATK signaling pathway (Chen et al., 2019). In addition, CLIC1 regulates migration and invasion in gastric cancer by triggering signaling of the ROS-mediated p38 MAPK pathway (Zhao et al., 2015). CLIC1 regulates colon cancer cell migration and invasion through ROS/ERK pathway (Wang et al., 2014). Thus, chloride flux may utilize distinct signaling pathways to execute specific functions in cellular context-dependent manners.

Beyond mammals, the biological roles of Cl^−^ in other species such as plants and nematodes have been sparingly studied (Chakraborty et al., 2017; Jentsch, 2008; Nguyen et al., 2016). For instance, in *Arabidopsis*, AtClC-e is localized to thylakoid membranes in the chloroplast (Marmagne et al., 2007), where its absence impairs the proton-motive force. Branicky et al. found that the CeClC-3 channel modulates the electrical activity of HSN neurons that control egg laying in *C. elegans* (Branicky et al., 2014). Overall, current knowledge on Cl^−^ function is mainly limited to mammals and tends to be more fragmentary concerning invertebrates. Despite substantial mammalian evidence that chloride channels are indispensable for robust phagosomal acidification and bactericidal activity (Jentsch, 2008; Moreland et al., 2006), how and to what extent chloride influx and chloride channels contribute to immune defenses in invertebrates is still under-examined. As a marine invertebrate with significant roles in ecological habitats, *Crassostrea gigas* has developed a versatile and intricate innate immune system capable of efficiently recognizing and removing invading pathogens (Wootton et al., 2003). From an evolutionary perspective, hemocytes in oyster are functional analogs of macrophages and neutrophils and are thus assumed to execute at least a subset of immune functions found in their human counterparts (Beaven and Paynter, 1999). Owing to a marine environment with high chloride, many physiological activities including host immune defense in oysters seem to be more dependent and susceptible to chloride than in terrestrial animals. In the present study, we set out to clarify the following cogent issues: (1) potential importance of chloride influx during phagocytosis of oyster hemocytes; (2) regulatory mechanisms that govern immune modulation by Cl^−^ influx; and (3) the cardinal chloride channel encoding gene that is responsible for Cl^−^ fluxes control in oyster hemocytes.

## Results

### Chloride Influx Is Activated during Phagocytosis

**Figure 1:**
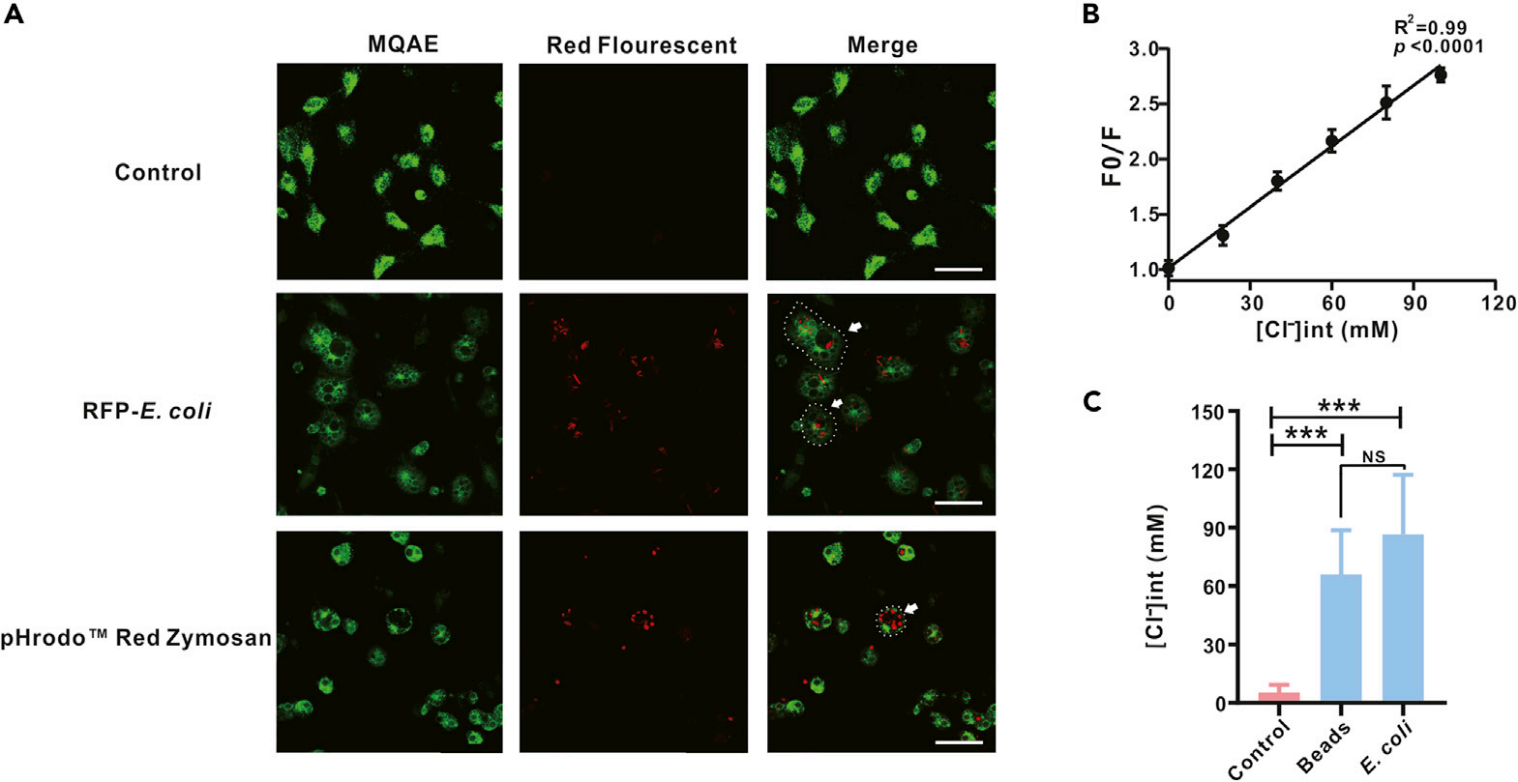
Chloride Influx Is Activated during Phagocytosis (A) Fluorescence of phagocytosed cells was significantly reduced. Oyster hemocytes loaded with MQAE (green) were challenged with pHrodo Red zymosan and fluorescent bacteria for 30 min at 27°C. MQAE is a fluorescent indicator that is quenched on encountering chloride. Cells were then imaged with a Leica SP8 inverted confocal microscope. White arrows indicate cells with significantly reduced fluorescence. Outline of cells pinpointed by an arrow is delineated by a dashed white line. MQAE signals of the experimental group were measured only in phagocytic cells. Scale bar: 10 μm. (B) Stern-Volmer calibration curve in HEPES-buffered standard solutions for MQAE (a Cl^−^-sensitive fluorescent dye) loaded into oyster hemocytes. Data are presented as mean ± SD. (C) [Cl^−^]_*i*_ in oyster hemocytes after phagocytosing beads and *E. coli* rose significantly. Fluorescence intensities of MQAE in oyster hemocytes at rest and hemocytes phagocytosing beads and *E. coli* were extracted to estimate [Cl^−^]_*i*_. Data were analyzed by using Image-Pro Plus 6.0. Data were analyzed by unpaired t test and presented as mean ± SD, ∗∗∗p < 0.001, NS: no significant difference, *n* = 3 groups, each group contains 10–15 cells.

To explore the possible immunodulatory roles of Cl^−^ influx in oyster hemocytes, we first examined whether Cl^−^ influx is activated during phagocytosis. Levels of intracellular Cl^−^ concentration ([Cl^−^]_i_) were measured by using the Cl-specific fluorescent probe MQAE. MQAE's fluorescence intensity decreases proportionally with increasing chloride ion concentration. Cell viability assay showed that hemocytes cultured *in vitro* keep a high cell viability under a broad range of temperature (Figure S1). Intriguingly, we observed a significant decrease in fluorescence intensity of MQAE (green Cl^−^ sensor) in phagocytes (red-fluorescence positive cells), upon hemocyte engulfment of either pHrodo Red zymosan or *E. coli* (Figure 1A), indicating an elevation of intracellular Cl^−^ concentration [Cl^−^]_*i*_ during phagocytosis. To calibrate [Cl^−^]_*i*_, a standard curve for MQAE fluorescence intensity versus [Cl^−^]_*i*_ was constructed by using a series of buffers prepared across a Cl^−^ concentration gradient (Koncz and Daugirdas, 1994) (Figure 1B). On the basis of no significant difference in the phagocytosis rate of pHrodo Red zymosan and *E. coli* (Figures S2A and S2B), calibrated by standard curve, [Cl^−^]_*i*_ was markedly increased from a baseline of 4.85 ± 4.49 to 65.74 ± 22.90 mM when hemocytes phagocytized zymosan-coated latex beads, and to 86.52 ± 30.71 mM in the case of *E. coli* (Figure 1C). However, no significant difference in magnitude of Cl^−^ influx was observed during phagocytosis whether for pHrodo Red zymosan or *E. coli*, suggesting that activation of Cl^−^ influx is a cellular event tightly coupled to phagocytosis initiation, regardless of the nature of phagocytosed substrates.

### Chloride Channel Inhibitor Blocks Phagocytosis in Hemocytes

**Figure 2:**
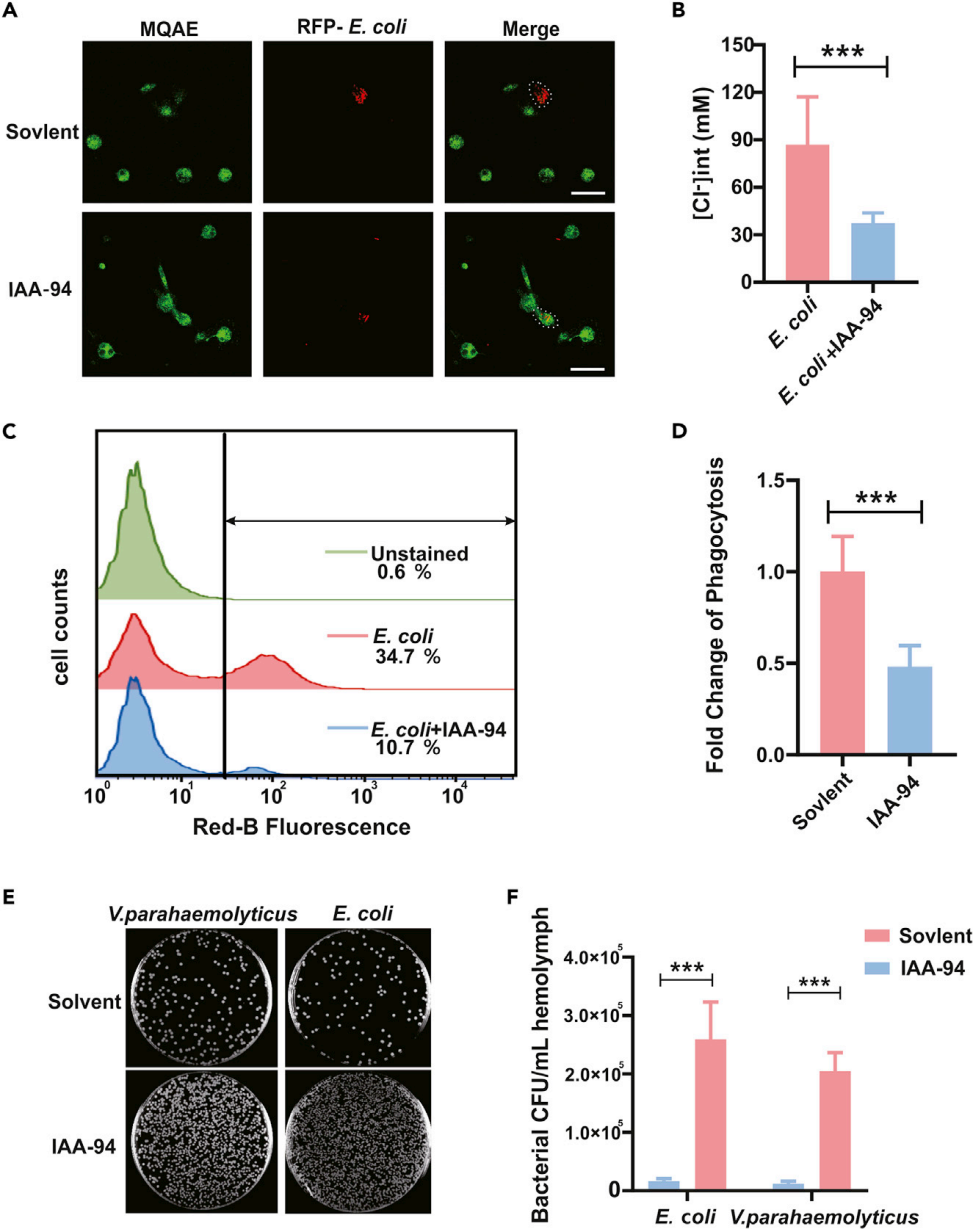
Chloride Channel Inhibitor Blocks Phagocytic Ability in Hemocytes (A) Fluorescence of IAA-94 treated phagocytes was enhanced. Oyster hemocytes engulfing fluorescent bacteria were loaded with MQAE (green). An intervention group was co-treated with IAA-94. Cells were imaged with a Leica SP8 inverted confocal microscope. Scale bar: 10 μm. IAA-94 is a potent indanyloxyacetic acid blocker of epithelial chloride channels. Outline of the cells is delineated by a dashed white line. MQAE signals were measured in phagocytic cells engulfing *E. coli*. (B) [Cl^−^]_*i*_ of IAA-94-treated phagocytes decreased. MQAE fluorescence intensities in oyster hemocytes treated with IAA-94 were extracted to estimate [Cl^−^]_*i*_. Data were analyzed by using Image-Pro Plus 6.0. Data were analyzed by unpaired t test and presented as mean ± SD, ∗∗∗p < 0.001, *n* = 3. (C) IAA-94 inhibited phagocytosis of *E. coli* by hemocytes. Under the premise that the number of cells in each group was approximately the same, flow cytometry analysis was conducted to gauge the extent of hemocytic phagocytosis. Red color represents the solvent-treated control and blue color represents the group treated with IAA-94. (D) Data analysis was performed by using GraphPad Prism 7 software. Data were analyzed by unpaired t test and presented as mean ± SD, ∗∗∗p < 0.001, *n* = 3. (E) IAA-94 inhibited bactericidal ability of hemocytes. Display of differences in bactericidal ability between the control group and the IAA-94-treated group. (F) Assay on bactericidal ability was analyzed by using GraphPad Prism 7. Data were analyzed by unpaired t test and presented as mean ± SD, ∗∗∗p < 0.001, *n* = 3.

To determine the function of Cl^−^ influx on phagocytic capacities of hemocytes, IAA-94, a potent indanyloxyacetic acid blocker of CLIC family channels, was employed in subsequent assays. In terms of intracellular Cl^−^ availability in oyster hemocytes, treatment of IAA-94 (100 μM) efficiently blocked infection-induced Cl^−^ influx activation (Figure S3), where the average steady-state [Cl^−^]_*i*_ of phagocytized hemocytes at 30 min post infection was dramatically reduced from 86.52 ± 30.71 to 36.33 ± 7.5mM, when compared with IAA-94 treatment group (Figures 2A and 2B). Meanwhile, the capacity of hemocyte to engulf bacteria was sharply reduced in the presence IAA-94, when compared with the basal control (treatment with solvent) (Figure S4). In agreement with this, the inhibitory effects of IAA-94 on hemocyte phagocytosis were confirmed in a quantitative manner by flow cytometry analysis (Figure 2C). The results suggest that IAA-94-treated group had an engulfment capacity approximately 50% less than that of the control group (Figure 2D). Understandably, the sequential processes of containment and killing of microbial pathogens are inseparable components of phagocyte-mediated defenses. Indeed, in bacterial killing assays, bactericidal capacity of hemocytes was greatly compromised after blockage of Cl^−^ influx. In contrast to the basal control, 30 min post infection, bacterial survival in IAA-94-treated hemocytes starkly increased (Figures 2E and 2F). Therefore, these observations strongly implicate that Cl^−^ influx is critical for phagocytic defense in oyster hemocytes through promoting capacity of engulfment and microbicidal activity.

### Chloride-Dependent Engulfment Is Promoted by PI3K/Akt Signaling Pathway

**Figure 3:**
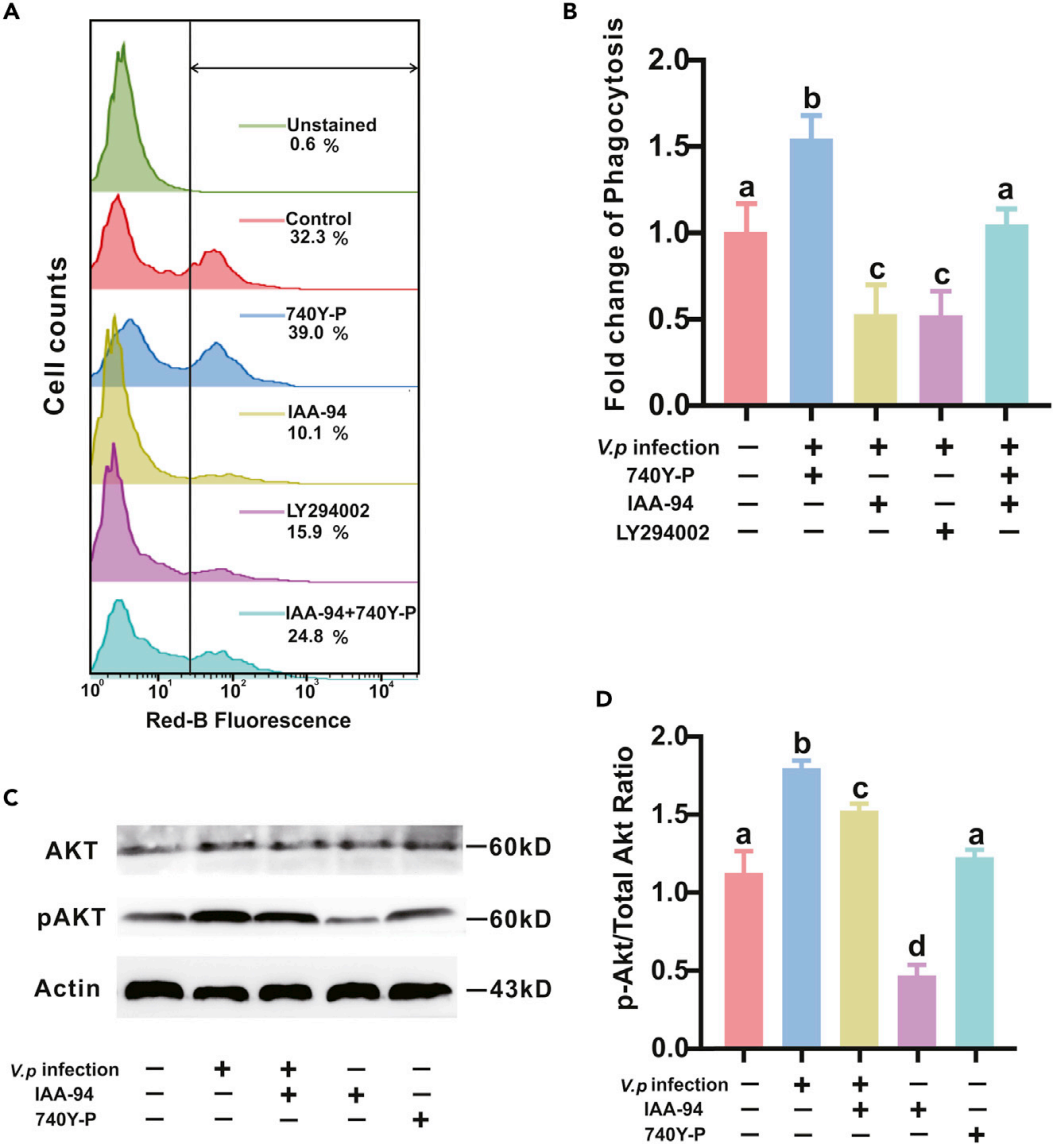
Chloride-Dependent Phagocytosis Is Regulated by PI3K/Akt Signaling Pathway (A) PI3K/Akt signaling pathway is involved in chloride-mediated phagocytosis. Flow cytometry analysis was used to detect hemocytic phagocytosis following bacterial infection in the presence or absence of pharmacological inhibitors as indicated. 740Y-P is a potent cell-permeable PI3K signaling activator. LY294002 is a broad-spectrum inhibitor of PI3K signaling. (B) Phagocytosis data analysis was performed by using GraphPad Prism 7 software. Data were analyzed by one-way ANOVA followed by Tukey's post hoc test, where p < 0.05 was considered to be statistically significant, as denoted by different letters. Data are presented as mean ± SD, *n* =3. (C) Western blot analysis on the effects of pharmacological intervention on bacterial infection: IAA-94 versus 740 Y-P with respect to the expression of proteins related to PI3K/Akt/mTOR signaling pathway. Levels of p-Akt/Akt was evaluated by western blot analysis. *β*-Actin served as a loading control. (D) p-Akt/Akt expression was measured densitometrically by using ImageJ. The data are normalized to *β*-Actin. Data analysis was performed by using GraphPad Prism 7 software. Data were analyzed by one-way ANOVA followed by Tukey's post hoc test, where p < 0.05 was considered to be statistically significant, as denoted by different letters. Data are presented as mean ± SD, *n* =3.

As a classical signaling pathway for regulating phagocytosis, the PI3K/Akt signaling pathway has been shown to be activated by chloride influx, leading to the hypothesis that PI3K/Akt signaling pathway may be a key regulator that controls or promotes Cl^−^-dependent phagocytosis in oyster hemocytes. As anticipated, phagocytic capacity of oyster hemocytes was significantly boosted (1.53-fold) upon treatment of a PI3K activator (740Y-P, 30 μM) compared with the control. Contrarily, phagocytic capacity was suppressed to 51% when hemocytes were co-treated with a PI3K inhibitor (LY294002, 1 μM), thus implicating the PI3K signaling pathway as an essential regulator for phagocytosis in oyster hemocytes (Figure 3A). More importantly, 740Y-P evidently rescued phagocytic impairments induced by IAA-94 in hemocytes, which raises the possibility of a functional interplay between PI3K/Akt signaling pathway and Cl^−^ influx mediating phagocytosis (Figure 3B). Furthermore, western blot analysis shows that the Akt phosphorylation increased substantially by 1.60- and 1.10-fold in infected and 740Y-P-treated oyster hemocytes, respectively, with respect to the control group. IAA-94 dampened Akt phosphorylation under both bacterial infection and untreated conditions, compared with the control group (Figures 3C and 3D). Taken together, these results support the notion that Cl^−^-dependent phagocytosis is likely promoted by the PI3K/Akt signaling pathway.

### Chloride Ion Flux Modulates Phagosomal Acidification and HOCl Production without Affecting Phagosomal-Lysosomal Fusion

**Figure 4:**
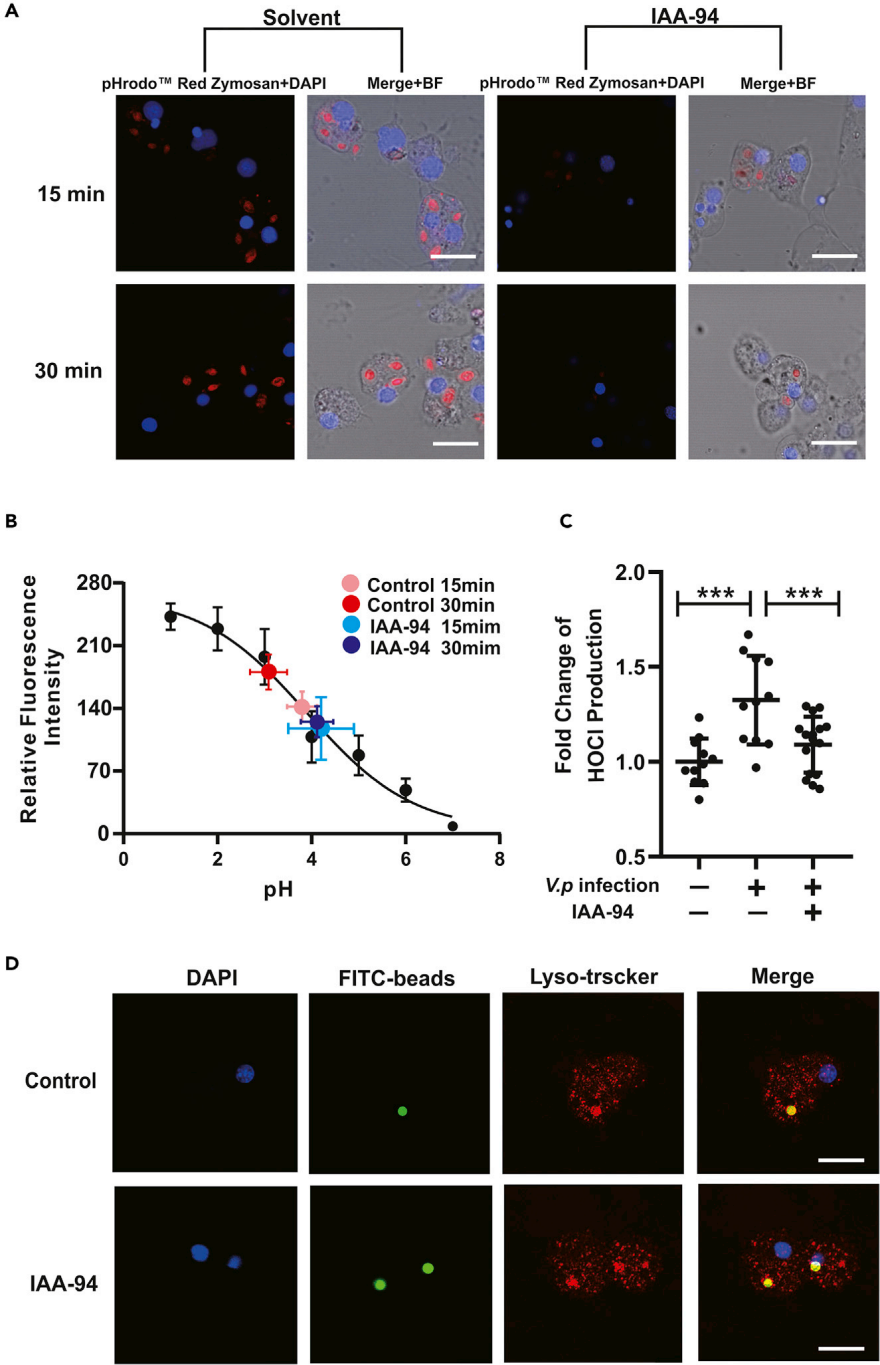
Chloride Flux Affects Phagosomal Acidification and HOCl Production but Not Phagosomal-Lysosomal Fusion (A) Acidification defects in phagosomes following particles ingestion in oyster hemocytes treated with IAA-94. Cells that had ingested pHrodo Red zymosan were observed by confocal microscopy. Scale bar: 5 μm. (B) Intraphagosome pH values are interpolated on a standard curve for pHrodo Red zymosan fluorescence versus pH determined *in vitro* by using a series of buffers. Fluorescence emission was calibrated and a fluorescence index was obtained from the samples (*n* = 3 groups, each group contains 10–15 cells). Bars represent as mean ± SD. (C) Following *in vivo* infection of oysters (*n* = 6 per group) with *V. parahaemolyticus*, in the presence or absence of IAA-94, HOCl production was assessed. Data analysis was performed by using GraphPad Prism 7 software. Data were analyzed by unpaired t test and presented as mean ± SD, ∗∗∗p < 0.01. (D) Representative confocal images of hemocytes during observations of phagosomal-lysosomal fusion 60 min from onset of phagocytosis. LysoTracker red is a red fluorescent probe for lysosomes that can be used for lysosome-specific fluorescent staining of living cells. Scale bar: 3 μm.

Since coordinated Cl^−^ ion flux is essential for pH regulation toward phagosomal acidification and catalytic production of HOCl, we examined whether these immunomodulatory roles of Cl^−^ are mechanistically conserved in the Pacific oyster. pHrodo Red dye (a fluorescent pH sensor) conjugated particles were used to determine any fluctuations in phagosomal acidification, whose signals show a negative correlation between its fluorescence intensity with pH (Figure S5). Following treatment of IAA-94 (100 μΜ), the intensity of phagosome-related fluorescence visibly declined at 15 and 30 min post phagocytosis (Figures 4A and 4B), which corroborates the assumption of Cl^−^ ion flux as an important modulator for phagosomal acidification in oyster hemocytes. To test whether additional factors can impede Cl^−^-dependent bacterial clearance in hemocytes, the hemocytic capacity for HOCl biosynthesis was assessed by an *in vivo* HOCl assay, with a standard curve constructed to calibrate the amounts of TNB versus optical density (HOCl levels) being determined (Figure S6). *V. parahaemolyticus* infection elicited 1.33-fold more HOCl, in comparison with the control group (Figure 4C), but this was abolished in the presence of IAA-94, implicating Cl^−^ ion flux as a significant regulator at work in infection-induced HOCl production. In addition, we also attempted to ask whether Cl^−^ ion flux impacts phagosomal-lysosomal fusion, as it is a critical step toward phagosomal maturation and bactericidal activity. However, our results show no appreciable differences between IAA-94-treated group and untreated group up to 60 min post phagocytosis (Figure 4D).

### CLIC2α Is a Primary Chloride Ion Channel within Oyster Genome

**Figure 5:**
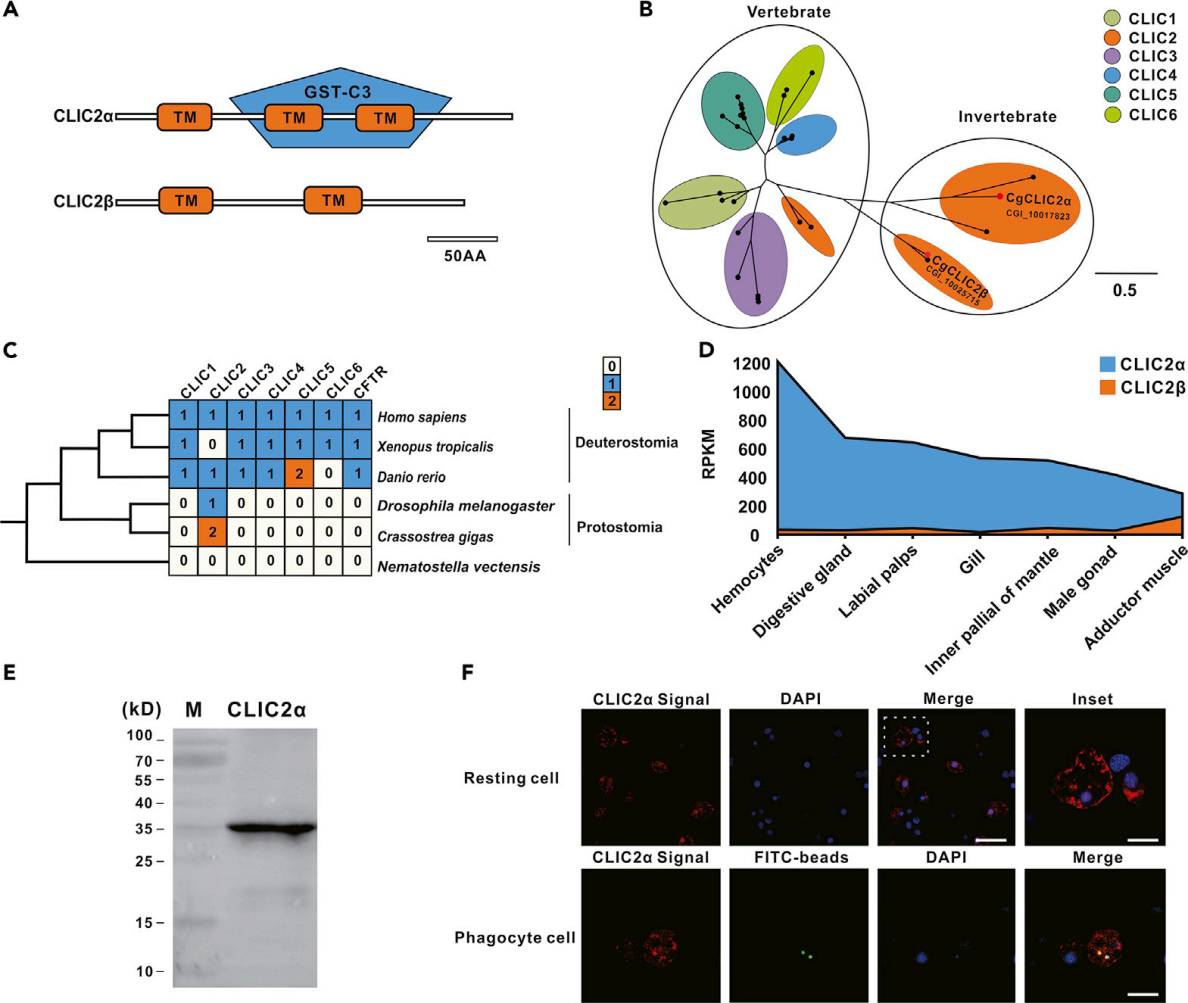
CLIC2α Is One of the Main Cl^−^ Channels in Oyster Genome and Subcellular Localization of CLIC2α in Oyster Hemocytes (A) Schematic representation of a comparison between transmembrane structures of intracellular Cl^−^ channel proteins in *C. gigas.* (B) CLIC superfamily protein sequences from different species including *Homo sapiens, Mizuhopecten yessoensis, Xenopus tropicalis, Danio rerio, Drosophila melanogaster*, and *C. gigas*. (C) Distribution patterns of six CLIC and CFTR genes. White, ortholog absent; blue, single-copy ortholog present; orange, double-copy ortholog present. (D) Analysis on expression levels of two intracellular Cl^−^ channel proteins in *C. gigas*. The *y* axis shows expression levels, and the *x* axis shows tissue sources. (E) Figure for western blot analysis of CLIC2α antibodies. (F) Immunofluorescence confocal micrographs show the spatial distribution of CLIC2α (red) as detected by a CLIC2α-specific primary antibody within oyster hemocytes. Nuclei (blue) were counterstained with DAPI. Scale bar: 5 μm. The insets show higher magnification. Scale bar of insets: 3 μm. Scale bar of phagocyte group: 4 μm.

To illuminate the genetic basis of Cl^−^ ion flux control in this ancient marine invertebrate, we set out to examine the gene families encoding the Cl^−^ channel in oyster and perform homologs search-based alignment on a genomic scale. Intriguingly, only two members of the CLIC family were identified in the Pacific oyster genome, whereas all CTFR homologs are absent (Figure 5A). A phylogenetic tree was then generated based on CLIC superfamily protein sequences from different species including *Homo sapiens*, *Mizuhopecten yessoensis*, *Xenopus tropicalis*, *Danio rerio*, *Drosophila melanogaster*, and *Crassostrea gigas*, which reveals that the CLIC family has highly diversified during evolution and considerably expanded in vertebrates, whereas the homologs of CTFR only prevail in vertebrate lineages (Figures 5B and S7). Based on the results of the evolutionary tree, we observed that CLIC2 is the only highly conserved Cl^−^ channel retained among ancient species such as *Protostomia* and *Deuterostomia*. Specifically, the ortholog of CLIC2 is duplicated in the genome of *C. gigas*, namely, CLIC2α and CLIC2β (Figure 5C). Tissue distribution showed that CLIC2α is predominantly expressed in hemocytes (Figure 5D), which suggests a key role of CLIC2α in regulating Cl^−^ transportation during hemocytes phagocytosis.

To further clarify the roles of hemocyte CLIC2α in immunological contexts, we attempted to determine the subcellular localization of CLIC2α by immunofluorescence. The antibody was verified by western blotting to ensure that the antibody specifically binds to the antigen stated (Figure 5E). Compared with non-immune lgG group, immunofluorescence showed that CLIC2α staining patterns were punctate or patchy within resting hemocytes, with evidently dense foci around the cell membrane in the resting hemocytes (Figures 5F and S8). Moreover, we also examined the subcellular localization of CLIC2α upon phagocytosis. The results show an evident overlay between CLIC2α positive signaling and FITC-beads (Figure 5F), implying phagosomal localization of CLIC2α in oyster hemocytes.

### Immunological Relevance of CLIC2α in Oyster Hemocytes

**Figure 6:**
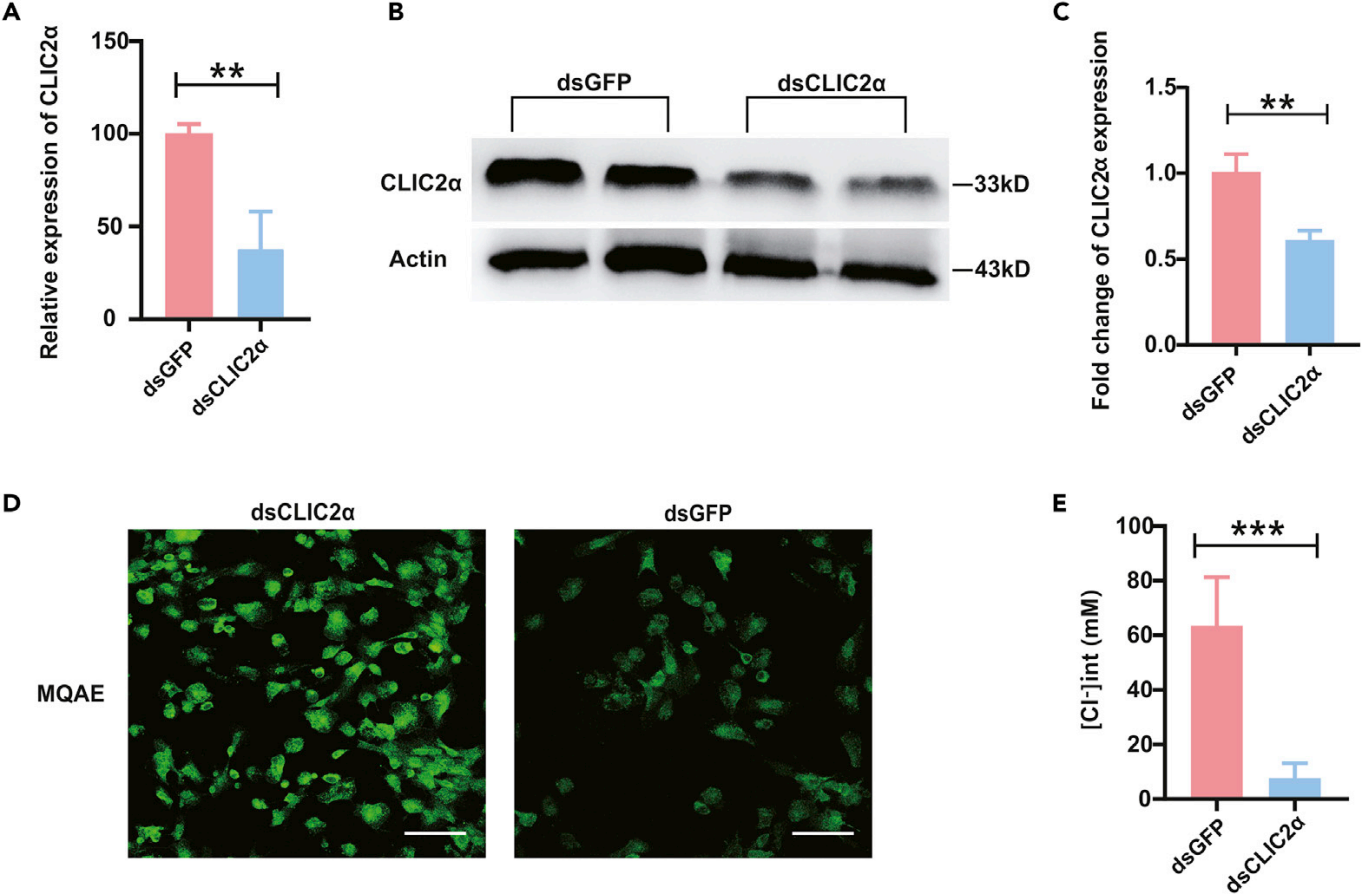
CLIC2α Is Responsible for Cl^−^-Mediated Innate Immunity in Oysters (A) Relative expression of CLIC2α is shown after RNAi in oyster hemocytes. Red color represents the dsGFP control group and blue color represents the dsCLIC2α group. mRNA levels were quantified by real-time PCR with GAPDH as a reference gene. Data were analyzed by unpaired t test and presented as mean ± SD, ∗∗p < 0.01, *n* = 3. (B) Western blot analysis on the effects of RNAi. Levels of CLIC2α was evaluated by immunoblotting. *β*-Actin served as a loading control. (C) CLIC2α expression was measured densitometrically by using ImageJ. Data analysis was performed by using GraphPad Prism 7 software. Data were analyzed by unpaired t test and presented as mean ± SD, ∗∗p < 0.01, *n* = 3. (D) Confocal micrographs show the fluorescence intensities of the dsCLIC2α and dsGFP groups of *Vibrio*-exposed hemocytes. Scale bar: 15 μm. (E) Data analysis was performed by using GraphPad Prism 7 software. Fluorescence intensities were normalized by cell number and volume of hemocytes. Data were analyzed by unpaired t test and presented as mean ± SD, ∗∗∗p < 0.001, *n* = 3.

To verify the function of CLIC2α as a Cl^−^ channel in oyster hemocytes, RNAi experiments were performed to knock down expression of CLIC2α. Results from quantitative PCR suggest that relative expression of CLIC2α was reduced by about 60% with respect to the dsGFP control group (Figure 6A), which is consistent with the knockdown efficiency at the protein level as verified by western blot (Figures 6B and 6C). As a test for validating Cl^−^ flux, fluorescence intensity of MQAE in the dsCLIC2α group was shown to be much enhanced compared with that of the dsGFP group in hemocytes under condition of *in vivo* infection by live *V. parahaemolyticus* (Figures 6D and 6E). Collectively, these observations confirm that CLIC2α is a conserved and functionally relevant Cl^−^ channel in oyster hemocytes.

**Figure 7:**
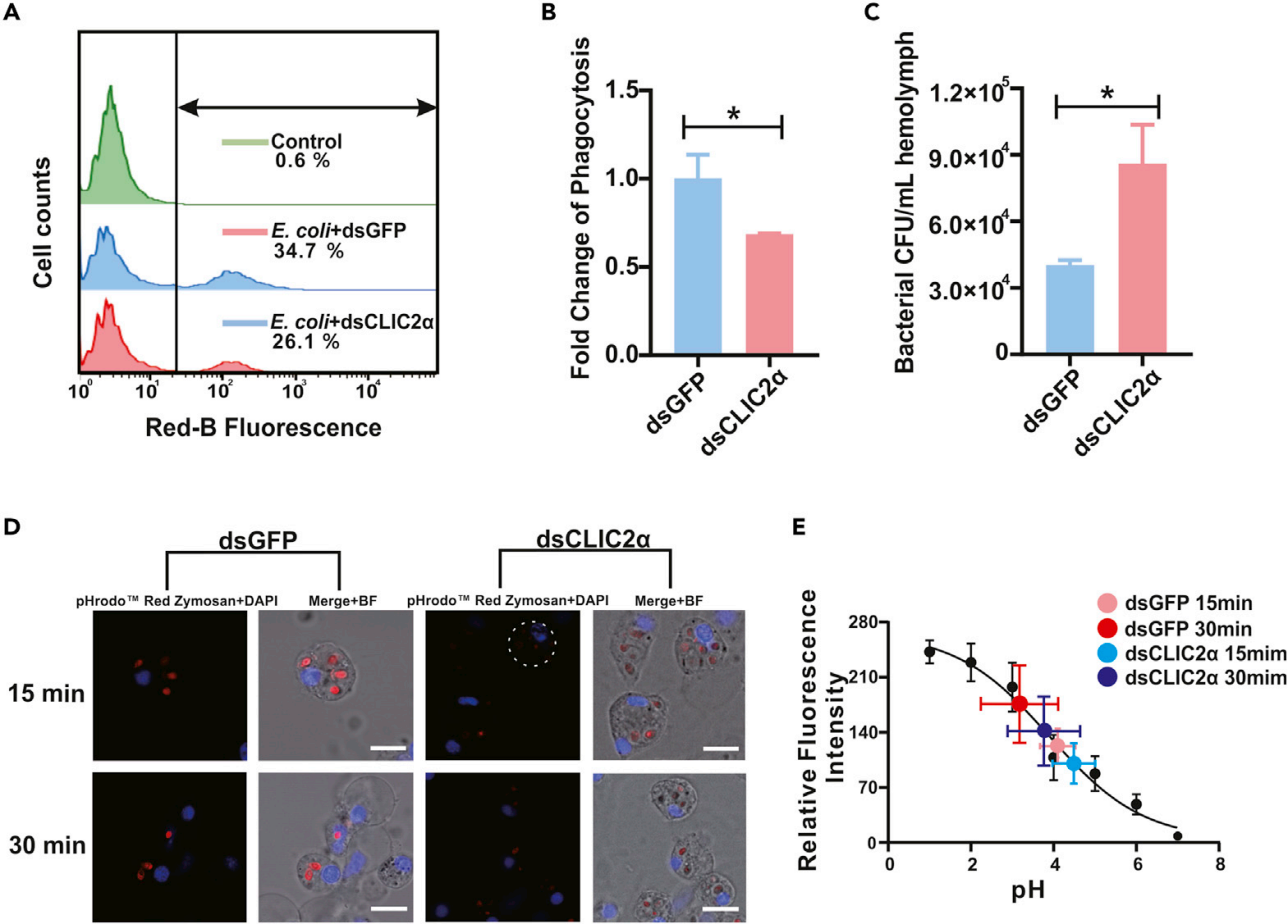
Immunological Relevance of CLIC2α in Oyster Hemocytes (A) Relative to the control group, ability of the dsCLIC2α group to swallow *E. coli* declined. Flow cytometry analysis was conducted to detect hemocytes phagocytosis of fluorescently labeled bacteria. Red color represents the dsGFP control group and blue color represents the dsCLIC2α group. (B) Data analysis for hemocytes phagocytosis was performed by using GraphPad Prism 7 software. Data were analyzed by unpaired t test and presented as mean ± SD, ∗p < 0.0, *n* = 3. (C) Relative to the control group, the bactericidal ability of the dsCLIC2α group decreased. Assay on bactericidal ability of the dsCLIC2α group and the dsGFP control group was analyzed by using GraphPad Prism 7 software. Data were analyzed by unpaired t test and presented as mean ± SD, ∗p < 0.05, *n* = 3. (D) Acidification defects in phagosomes following particle engulfment in oyster hemocytes silenced by dsCLIC2α, in comparison with dsGFP group. Cells that had ingested pHrodo Red zymosan were observed by confocal microscopy. Scale bar: 5 μm. (E) Fluorescence emission was calibrated and a fluorescence index was obtained from the samples (*n* = 3 groups, each group contains 10–15 cells). Bars represent mean ± SD.

As CLIC2α is known to serve as a Cl^−^ channel that controls the flow of Cl^−^ ion into the cytosol, it is reasonable to conclude that CLIC2α resides on the hemocytic cell membrane to exercise this function. Given the abundance of CLIC2α in hemocytes (Figure 5D), we reasoned that CLIC2α protein is responsible for Cl^−^-mediated phagocytic defenses during *in vivo* infection by live *V. parahaemolyticus*. Quantitative analysis by flow cytometry (Figure 7A) shows that CLIC2α knockdown resulted in a reduction of 32% in phagocytic capacity of hemocytes compared with the control group (Figure 7B). Bacterial survival counts of infecting *V. parahaemolyticus* rose from 3.98 × 10^4^ to 8.55 × 10^4^ CFU following RNAi of CLIC2α (Figure 7C). Furthermore, knockdown of CLIC2α in hemocytes clearly compromised their ability to acidify phagosomes (Figures 7D and 7E), which likely has adverse consequences for bacterial clearance. Overall, these results implicate RNAi of CLIC2α with impaired hemocytic phenotypes in phagocytosis, phagosomal acidification, and microbial killing, thus supporting an essential role of CLIC2α in Cl^−^-mediated innate immunity in oysters.

## Discussion

Chloride ion is a ubiquitous yet indispensable constituent in phagocyte biology. It is responsible for mediating numerous physiological functions including maintenance of membrane potential, regulation of phagosomal pH, promotion of phagosomal enzymatic activities, and production of Cl^−^-containing oxidants as antimicrobial agents (Wang, 2016). Chloride ion channels (CLICs) have been intimately linked to inflammatory diseases, inspiring enduring interest in their immune roles (Gururaja Rao et al., 2020). Despite substantial advances in vertebrates, mechanistic understanding on the immune function of Cl^−^ in invertebrates remains scant. Here, we have revealed for the first time that coordinated phagocytic events associated with Cl^−^ flux occur after infection and that an under-examined Cl^−^ channel, CLIC2α, plays vital roles in promoting hemocyte phagocytosis and bactericidal activity of *C. gigas*, a representative species in lower marine invertebrates.

Functional diversity abounds in the regulation and direction of Cl^−^ flow/flux across different cell types. For instance, intracellular Cl^−^ concentration was markedly elevated in human airway epithelial BEAS-2B cells upon challenge with LPS (lipopolysaccharide) from *P. aeruginosa* (Zhang et al., 2018). In a similar case, [Cl^−^]_*i*_ of resting oyster hemocytes is kept at a low level but surges upon infections. In contrast, Cl^−^ efflux ensued when human neutrophils were exposed to *Candida albicans* opsonized with various soluble factors (Busetto et al., 2007). The reason for the different flow directions of Cl^−^ is uncertain and may concern how different cells interact with exogenous danger signals. Regardless, productive acidification of phagosomes is invariably a mandatory requirement for proper phagocytic functions. Phagosomal pH is primarily controlled by activity of H^+^-pumping vacuolar-type ATPases (V-ATPase) and importation of anions (Kissing et al., 2018). Proton pumping, which depresses luminal pH, generates an electrochemical potential difference across membranes (positive charge on the luminal side). If left unchecked, this driving force would begin to decelerate and eventually inhibit further acidification (Grabe et al., 2000; Kettner et al., 2003). To prevent premature inhibition, the generated voltage is offset by import of anions (Cl^−^) and/or export of cations (Kissing et al., 2018). As a result, inappropriate or insufficient Cl^−^ flux can be an important cause of impaired phagosomal acidification. With the efflux of Cl^−^ ions, neutrophil phagosomes initially alkalinize and slowly acidify to a moderate level (El Chemaly et al., 2014; Jankowski et al., 2002). Additionally, ClC3 chloride channel is reportedly expressed in neutrophils and their phagosomes, modulating phagosomal pH (Scheel et al., 2005). Deletion of CFTR in murine alveolar macrophages caused defective phagosomal acidification and bactericidal activity (Deriy et al., 2009; Di et al., 2006). Consistent with these previous findings, we have demonstrated blocking of Cl^−^ flux disrupts phagosome acidification and thus its phagocytic function in oyster hemocytes.

An acidic environment exerts modulatory effects on the structural maturation of hydrolases and denaturation of their protein substrates, which ultimately increases the rates of killing engulfed bacteria (Pillay et al., 2002). For complementing this mode of antibacterial defense, phagocytes also use a combination of oxidative mechanisms, including the production of superoxide (O_2_^•-^) and hypochlorous acid (HOCl), to eradicate phagocytosed microorganisms (Kobayashi et al., 2000). Superoxide produced in phagocytes is unstable and has only mild antimicrobial potential. Instead of directly leveraging O_2_^•-^, phagocytes convert it to hydrogen peroxide (H_2_O_2_), a long-lasting oxidant with modest antimicrobial activity at millimolar level (Bonvillain et al., 2011; Lymar and Hurst, 1995). Subsequently, H_2_O_2_ is turned into the much more potent HOCl via MPO (Bonvillain et al., 2011). Evolutionarily, regulated production of ROS such as HOCl represents a major advance in phagocytic innate immunity (Albrich et al., 1981). In this study, we found that oysters have evolved an efficient machinery to produce these oxidizing biocides. Under conditions of bacterial stimulation, production of HOCl increased, whereas blocking Cl^−^ flux resulted in its inhibition.

Apart from implications in ROS production, Cl^−^ flux seems to mediate hemocytic functions in PI3K/Akt-dependent manners. Previous research findings have shown that PI3K plays an indispensable role in phagosome formation and maturation. During initial phagocytosis, PI3K controls actin depolymerization to drive the formation of a phagocytic cup that surrounds a foreign body and bring it into the phagosome (Olazabal et al., 2002; Tuxworth et al., 2001). Pharmacological inhibition of PI3K could impair the ability of phagocytosis (Araki et al., 1996, 2003; Cox et al., 1999). Moreover, PI3K/Akt/mTOR signaling regulates phagosome maturation by modulating microtubule-based motor activity to modulate events of lysosome trafficking and fusion in leukocytes (Saric et al., 2016). Consistently, our study confirms that PI3K/Akt signaling in the regulation of phagocytosis is conserved from oyster to mammals. More importantly, the PI3K/Akt pathway is crucial to mediating Cl^−^ flux-dependent phagocytosis, which is supported by the evidence that the PI3K activator 740Y-P could rescue blockage of phagocytosis caused by chloride channel inhibitors IAA-94. However, the chloride channel inhibitors IAA-94 had no effects on the fusion of lysosomes and phagosomes as reported in macrophages (Jiang et al., 2012), which seems to contradict with the anticipated roles of PI3K/Akt in phagosome maturation. One possibility is that Cl^−^ flux may activate other signaling pathways or regulatory mechanisms of intracellular feedback to brake on the fusion of lysosomes and phagosomes, which remains to be verified.

In this study, phylogenetic analysis with functional validation showed that CLIC2α is the primary Cl^−^ channel in oyster, and we thoroughly investigated the potential roles of the Cl^−^ channel CLIC2α in innate immunity in oyster hemocytes. CLIC2α was identified as a CLIC family member protein with the highest expression levels in the oyster. CLICs are highly conserved in chordates with six vertebrate paralogs, from CLIC1 to CLIC6. According to the phylogenetic tree, only CLIC2 exists in invertebrates and expands to CLIC2α and CLIC2β. From an evolutionary perspective, CLIC2 appears to be the only one Cl^−^ channel with function conserved from an ancestral progenitor of *Protostomia* and *Deuterostomia*. Subcellular localization of one specific protein is generally associated with its function and also provides clues on regulatory mechanisms. In mammalian macrophages, CLIC1 occurs as spots in the cytoplasm and is translocated to the phagosomal membrane during phagocytosis (Jiang et al., 2012). In contrast, CLIC2α in oysters is mainly localized in the cellular membrane but could be translocated to the phagosomal membrane in manners resembling macrophage CLIC1. Given that composition-wise the phagosomal membrane originates from the plasma membrane, membrane trafficking may be one possible route for CLIC2α translocation. Furthermore, knocking down CLIC2α evidently diminished Cl^−^ flux and disrupted Cl^−^-dependent phagocytic functions in oyster hemocytes, including phagocytosis, phagosomal acidification, and bacterial killing. Beyond the current scope of investigation on phagocytic immunity, it is quite likely that CLIC2α performs other biological functions central to cell physiology in vertebrates and invertebrates alike. Future studies on novel CLIC2α functions and their underlying mechanisms are thus warranted.

### Limitations of the Study

In this study, we have explored the subcellular localization and function of CLIC2α mainly in bacterial infection contexts. Further in-depth characterization of the exact roles of CLIC2α in governing the downstream of PI3K/Akt or other intracellular signaling pathways in immune defense and how CLIC2α could be activated would be pertinent.

### Resource Availability

#### Lead Contact

Further information and requests for resources should be directed to and will be fulfilled by the Lead Contact, Yang Zhang (yzhang@scsio.ac.cn).

#### Materials Availability

All unique/stable reagents generated in this study are available, on reasonable request, from the Lead Contact on a completed Materials Transfer Agreement.

#### Data and Code Availability

All data supporting the findings of this study are included in the article and its Supplemental Information or are available from the corresponding authors on request.

## Methods

All methods can be found in the accompanying Transparent Methods supplemental file.

## Supplemental Information

Document S1. Transparent Methods, Figures S1–S8, and Table S1

## Footnotes

Supplemental Information can be found online at https://doi.org/10.1016/j.isci.2020.101328.
