# Supplementary material for: CLIC2α Chloride Channel Orchestrates Immunomodulation of Hemocyte Phagocytosis and Bactericidal Activity in Crassostrea gigas
Source: iScience. 2020 Jun 30;23(7):101328. doi: 10.1016/j.isci.2020.101328 (PMC7363696; doi:10.1016/j.isci.2020.101328)
Supplement: Document S1. Transparent Methods, Figures S1–S8, and Table S1 [file mmc1.pdf]

iScience, Volume *23*

Supplemental Information

CLIC2α Chloride Channel Orchestrates Immunomodulation of Hemocyte Phagocytosis and Bactericidal Activity in *Crassostrea gigas*

Xiangyu Zhang, Fan Mao, Nai-Kei Wong, Yongbo Bao, Yue Lin, Kunna Liu, Jun Li, Zhiming Xiang, Haitao Ma, Shu Xiao, Yang Zhang, and Ziniu Yu

Figure S1.

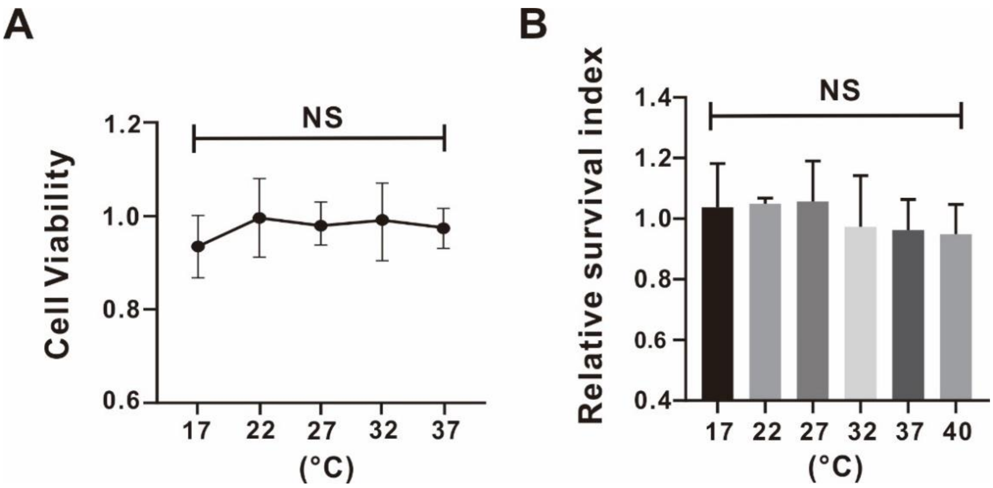

Figure S2.

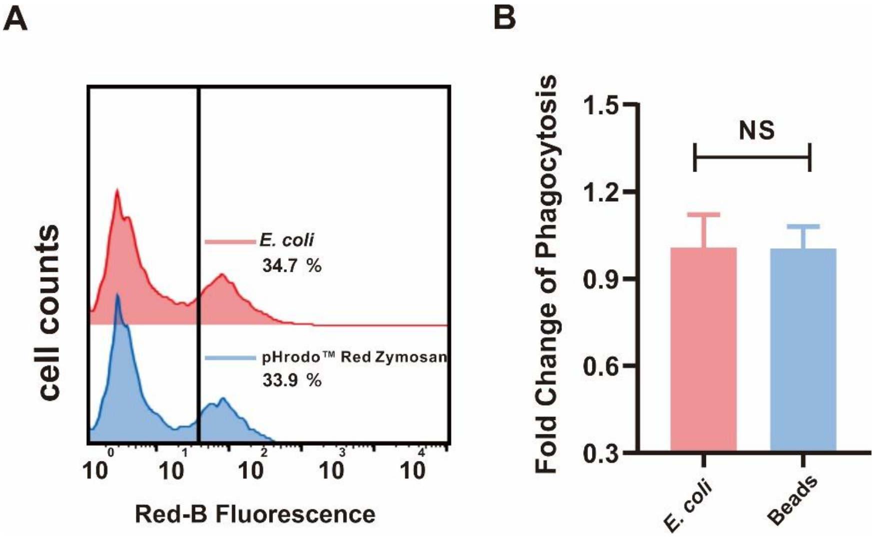

Figure S3.

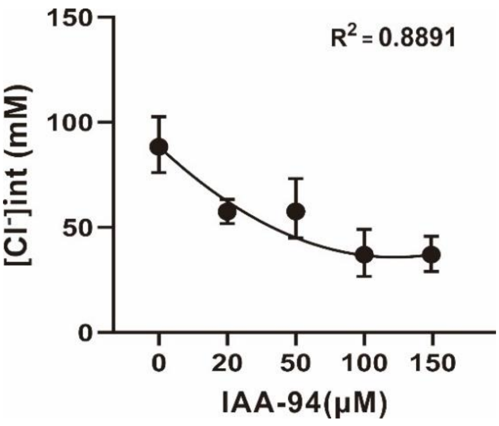

Figure S4.

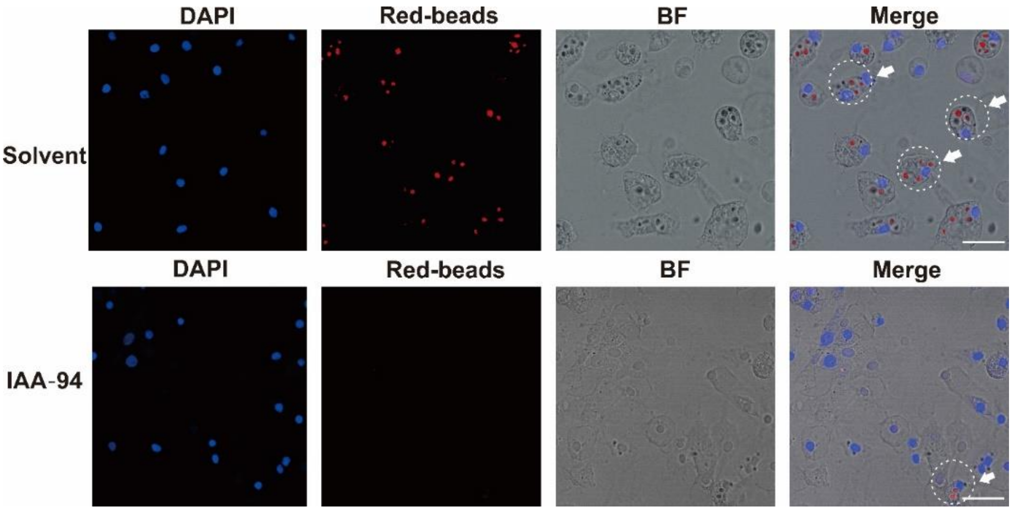

Figure S5.

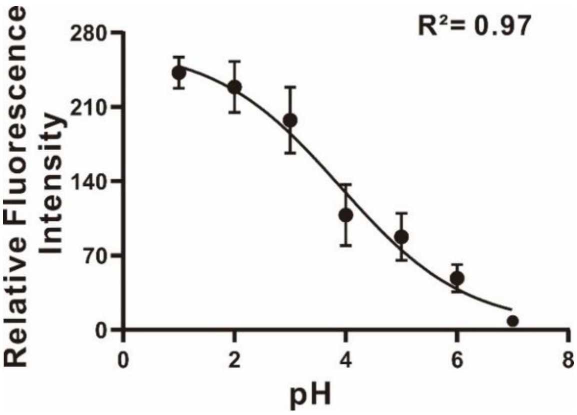

Figure S6.

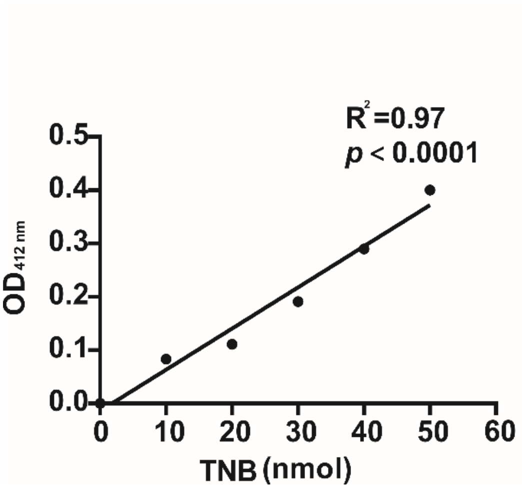

Figure S7.

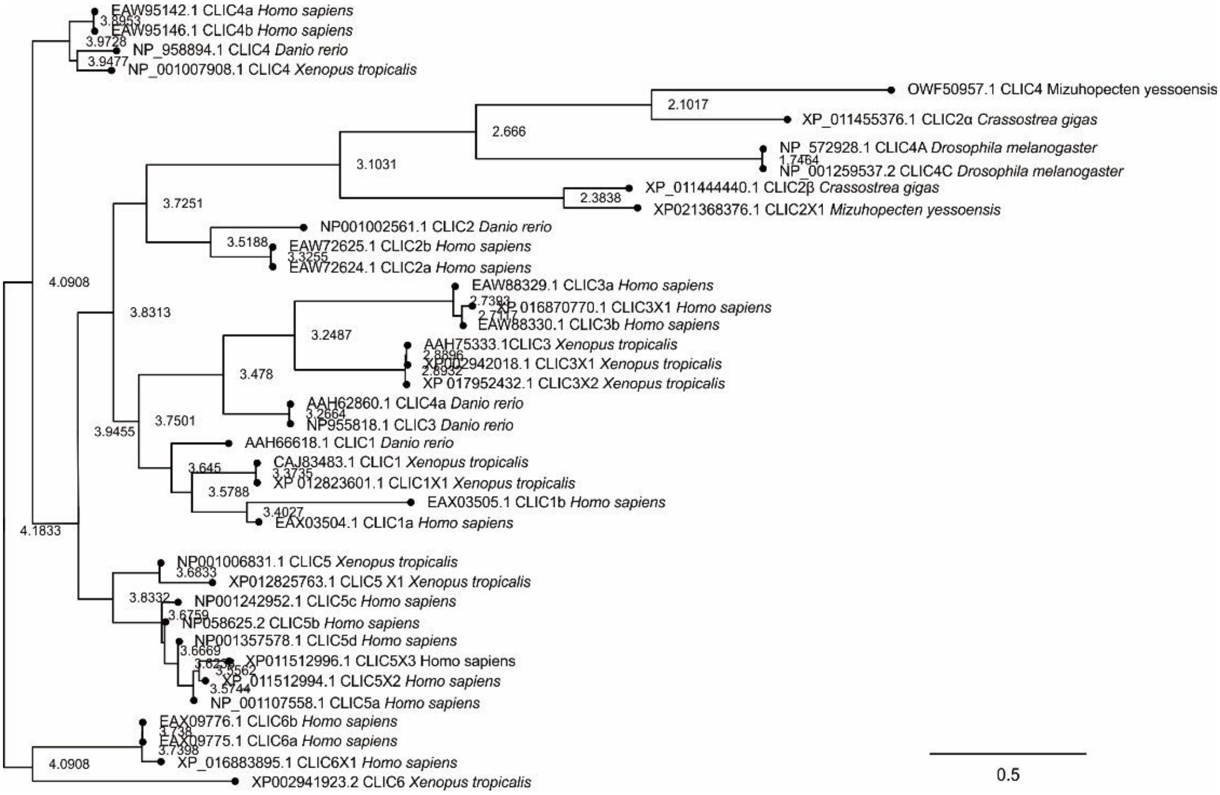

Figure S8.

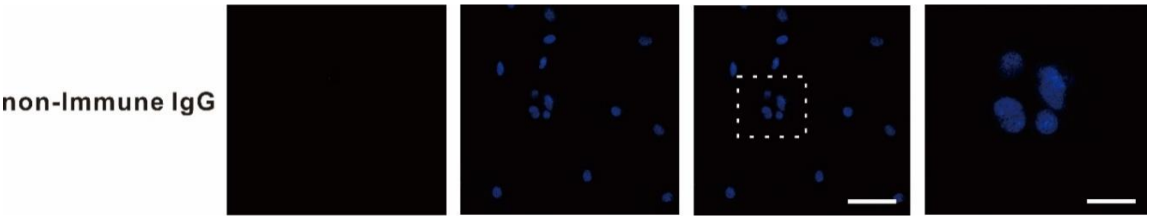

Supplemental Figure Legends

Figure S1. Cell viability tests of oyster hemocytes during 2 h of *in vitro* primary culture, related to Figure 1. A, Cells viability was assessed by measurement intracellular ATP levels. B, Relative cell survival rates were measured by Trypan blue staining. Statistical analysis was done by one-way ANOVA followed by Tukey's *post hoc* test; NS: no significant difference, all data are presented as mean ± S.D. (n = 3).

Figure S2. Flow cytometry analysis was conducted to measure the rate of phagocytosis, related to Figure 1. A, Red color and blue color represent groups phagocytosing *E. coli* and pHrodo™ Red zymosan, respectively. B, Data analysis was performed by using GraphPad Prism 7 software. n = 3; data were analyzed by unpaired t test, and asterisks in the graphs

indicate *p* values of significance; NS: no significant difference; data are presented as mean ± S.D.

Figure S3. Dose-dependent response curve for IAA-94 effects on chloride flux in oyster hemocytes after bacterial challenge, related to Figure 2. IAA-94 is a potent indanyloxyacetic acid blocker of epithelial chloride channels. All data are presented as mean ± S.D. (n = 3).

Figure S4. Dysphagocytosis in phagosomes following particles ingestion in oyster hemocytes treated with IAA-94, related to Figure 2. Arrows refer to cells that have engulfed the beads.

Figure S5. Analysis on the relationship between fluorescence intensity and pH of pHrodo™ Red zymosan, related to Figure 4. All data are presented as mean ± S.D. (n = 3).

Figure S6. Standard curve for TNB (measure of production of hypochlorous acid) and optical density, related to Figure 4.

Figure S7. CLIC superfamily protein sequences from different species including *Homo sapiens, Mizuhopecten yessoensis, Xenopus tropicalis, Danio rerio, Drosophila melanogaster and Crassostrea gigas*, related to Figure 5. Protein sequence accession numbers are as shown at the end of branch name.

Figure S8. Negative controls for subcellular localization of CLIC2α, related to Figure 5. Negative control groups are stained with non-immune IgG. Nuclei (blue) were counterstained with DAPI. Scale bar: 5 μm. Insets show higher magnifications. Scale bar of insets: 3 μm.

Supplemental Table

Table S1. Summary of primers used in this work, related to Figure 6 and Figure 7.

Names	Sequences (5'-3')
DsRNA templates amplification	
DsCLIC2α-F	TAATACGACTCACTATAGGTCCCAGTAGTGATTGGAACGAG
DsCLIC2α-R	TAATACGACTCACTATAGGCCCTGTATTGCTCTCCTGCC
DsGFP-F	GGATCCTAATACGACTCACTATAGGACAAGTTCAGCGTGTCCG
DsGFP-R	GGATCCTAATACGACTCACTATAGGTTCACCTTGATGCCGTTC
Quantitative RT-PCR (qRT-PCR)	
Q-CLIC2α-F	TATGGAACGTGGATACCAGACAA
Q-CLIC2α-R	CCGTTTGACCCTTCACCATC
Q-GAPDH-F	GGATTGGCGTGGTGGTAGAG
Q-GAPDH-R	GTATGATGCCCCTTTGTTGAGTC

Transparent Methods

Animals, pathogen challenge and hemolymph preparation

Crassostrea gigas (2 years old) were obtained from the Marine Biology Research Station at Zhanjiang of the Chinese Academy of Sciences (Zhanjiang, Guangdong, China). For acclimation, oysters were maintained at 27±1°C in tanks with re-circulating seawater for one month before experiments. Oysters were fed twice daily with *Tetraselmis suecica* and *Isochrysis galbana*. All animal experiments were conducted in accordance with the guidelines and approval of Animal Research and Ethics Committees of the Chinese Academy of Sciences.

For bacterial challenge, *Vibrio parahaemolyticus* was cultured in LB (Luria-Bertani) broth at 37°C to a turbidity of OD_{600nm} = 0.6-0.8, and then centrifuged at 800×g for 10 min at 4°C. After being washed for 3 time in PBS (PBS, 0.14 M sodium chloride, 3 mM potassium chloride, 8 mM disodium hydrogenphosphate dodecahydrate, 1.5 mM potassium phosphate monobasic, pH 7.4), bacterial pellet was resuspended in PBS to an adjusted density of OD_{600nm} = 1.0. Oysters in the challenged group were injected with a 100-μL suspension into the adductor muscle, while oysters in the control group were injected with an equal volume of PBS. After injection, oysters were returned to separate tanks for subsequent incubation or sampling.

Hemolymph was drawn from the posterior adductor muscle of *C. gigas* by using a sterile 1-mL syringe. Hemolymph was kept and immediately centrifuged at 200×g for 10 min to collect hemocytes, then the supernatant was sterilized through a 0.22 μM filter (Sangon Biotech, F513134). Hemocytes were incubated in each petri dish with hemolymph serum at 27°C for subsequent analyses.

Cell viability test

Approximately 1×10^6 cells were cultured in 12-well plates with filtered plasma and incubated at different temperatures. CellTiter-Lumi™ Luminescent Cell Viability Assay Kit (Promega, USA) was used to quantify ATP content, which is one marker for living cells. According to the manufacturer's protocol (Beyotime, C0065S), treated hemocytes were equilibrated at room temperature for 10 min for the next operation. 50 μL CellTiter-Lumi ™ luminescence detection reagent was added to each well of a 96-well microplate. The 96-well microplate was gently shaken at room temperature for 2 min to promote cell lysis. Lysed cells were incubated at room temperature (about 27°C) for 10 min to stabilize luminescence signals. A multi-functional microplate reader was used for chemiluminescence detection of samples. Relative viability of cells was directly calculated based on chemiluminescence readings.

In Trypan blue staining assay, hemocytes were stained with a standard Trypan blue solution (0.04% in PBS) for 5 min. Cells were enumerated under a light microscope with a hemocytometer, where blue-colored cells were considered to be dead. All experiments were performed in at least triplicates.

Determination of $[Cl^-]_i$

This part of the study was conducted as previously described with slightly modification

(Zhang et al., 2018). Briefly, levels of intracellular Cl^- concentration ($[Cl^-]_i$) were measured by using the Cl^- specific fluorescent probe MQAE (5 mM, Beyotime, S1082), a selective chloride ion indicator. Upon binding halide ions such as chloride, MQAE fluorescence is quenched, resulting in a decrease in fluorescence intensity without a shift in wavelength (Ikeuchi et al., 2018).300 μL of hemocytes were cultured in glass bottom dishes at 27°C for 15 min. At the end of appropriate treatments, oyster hemocytes were washed twice with Cl^--free Tyrode solution (NaCl was replaced by equimolar amounts of *D*-glucuronic acid; $MgCl_2$ by $MgSO_4$; KCl by potassium gluconate), and loaded with 10 mM of MQAE in dark at 27°C for 30 min Thereafter, samples were rinsed 3 times with Cl^--free Tyrode solution. To calibrate the probe's fluorescence intensities relative to $[Cl^-]_i$, standard Tyrode solutions are configured and delivered as described. Different predetermined concentrations of Cl^- (20, 40, 60, 80 and 100 mM) were prepared with the solutions which, where appropriate, also contained tributyltin chloride (10 μM, J&K Scientific, T773810), nigericin (10 μM, MedChemExpree, HY-127019) and valinomycin (10 μM, MedChemExpree, HY-N6693) to disrupt membrane channel activities and allow equilibration of intracellular Cl^- with that of an extracellular buffer. Then, a calibration curve was constructed by fitting fluorescence intensities onto corresponding Cl^- concentrations, and $[Cl^-]_i$ could be calculated based on the calibration curve.

For other experiment, the oyster hemocytes were cultured with filtered plasma, and loaded with 10 mM of MQAE in dark at 27°C for 30 min for florescence detection. IAA-94 (100 μM, MedChemExpree, HY-12693), a cell permeable chloride ion channel blocker, or DMSO (vehicle) was treated to determine changes in $[Cl^-]_i$ during phagocytosis in oyster hemocytes.

Phagocytosis assay

E. coli transformed with an indicator plasmid (pFPV25.1) expressing RFP (red fluorescent protein) was cultured to a turbidity of OD_{600nm} = 0.6-0.8 at 37°C. Then, the bacterium was washed 3 times in PBS (0.14 M sodium chloride, 3 mM potassium chloride, 8 mM disodium hydrogenphosphate dodecahydrate, 1.5 mM potassium phosphate monobasic, pH 7.4) and resuspended to a final density of 1.0×10^7 CFU per mL for subsequent phagocytosis assays (Duperthuy et al., 2011; Wang et al., 2014). Hemocytes cultured in a 24 well plate and the determined reagent were included in the buffer to incubate the cells for 30 minutes at room temperature and washed 3 times with PBS. Next, hemocytes were incubated with the prepared bacteria for 15 min at a MOI (multiplicity of infection) of 50. Cell were washed with Tris buffer (pH 8.0, 50 mM, Sangon Biotech, A610195) for 3 times to remove unbound bacteria and then suspended in PBS supplemented with 1.5‰ EDTA. Trypan blue was used to inhibit further attachment of hemocytes to bacteria (Guckian et al., 1978). Finally, flow cytometry analysis by Guava® easyCyte™ was performed to quantify phagocytosis in oyster hemocytes. Cells taking up *E. coli* were recognized by RFP reporter fluorescence, which provided an indication of uptake capacity (proportional to the number of bacteria retained). Phagocytosis by hemocytes was monitored with at least a total of 10,000 events. Data were analyzed with the FlowJo

software (version V10).

Bacterial clearance assay

This assay was performed as described previously with minor modifications (Saleh et al., 2006). For the experiments, two strains of bacteria, *Escherichia coli* (DH5α) and *V.parahaemolyticus* (ZJ51) (of a working density at OD_{600nm} = 0.2), were cultured at 37°C. After incubation, bacteria were harvested by centrifugation at low speed, followed by washing 3 times with Tris buffer (50 mM, pH 8.0, Sangon Biotech, A610195) and resuspension in 1 ml PBS for subsequent assays. Approximately 2×10^5 hemocytes per well was cultured in a 24-well plate and subsequently challenged with preprocessed bacteria at an MOI (multiplicity of infection) of 50 at room temperature. Cells were then briefly treated with 0.02% trypsin-EDTA for 4 times to remove extracellular bacteria. Subsequently, IAA-94 (100 μM) and DMSO (vehicle) were added to the filtered body fluids (except for the knockdown group) to pretreat cells for 30 min. After 30 min to kill the internalized bacteria, hemocytes were lysed in 1 mL PBS containing 0.05% Triton X-100. Finally, 100 µL of the lysate was used as an inoculum on LB agar plates for enumerating bacterial colonies. For each group, three wells were used to perform the bacterial clearance assay and each experiment was independently repeated 3 times.

Western blot analysis

Hemocytes were collected and each sample was analyzed in triplicates. Specifically, hemocytes were harvested and lysed in IP buffer supplemented with a protease and phosphatase inhibitor cocktail according to the manufacturer's protocol (Sangon Biotech, C50035). Lysates were centrifuged at 1,200×g for 20 min at 4°C, and supernatant was diluted 10 times to determine protein concentrations by using the bicinchoninic acid (BCA) protein assay (ThermoFisher Scientific, 23227). Samples containing equal amounts of proteins per lane were resolved by sodium dodecyl sulfate-polyacrylamide gel electrophoresis (SDS-PAGE) and then transferred to 0.2 μm polyvinylidene difluoride membranes (Merck Millipore, ISEQ00010). The membranes were blocked with QuickBlock™ blocking buffer for Western blotting (Beyotime, P0252), and then incubated with appropriate primary antibodies overnight at 4°C. The membranes were washed with PBST (phosphate buffered solution with 0.1% Twen-20 [Damao, 9005-65-6]), and then incubated for 2 h at room temperature with a secondary antibody. All primary and secondary antibodies used were diluted by a ratio of 1:1000 and 1:2000, respectively, in QuickBlock™ blocking buffer. The protein bands of interest were visualized by using an ECL luminescence reagent (Sangon Biotech, C510043). Relative expression of protein was quantified by using ImageJ software (version 1.8.0).

The following antibodies were used to detect their corresponding protein substrates: rabbit anti-Akt (Cell Signaling Technology, C67E7), rabbit anti-phospho-Akt (Thr308) (Cell Signaling Technology, 244F9), rabbit anti-*β*-actin (Cell Signaling Technology, 8457), anti-rabbit HRP-linked secondary antibody (Cell Signaling Technology, 7074).

Determination of phagosomal acidification index

Freshly harvested hemocytes were incubated in 35-mm diameter glass-bottom dishes (NEST, 801001) for 30 min at 27°C with pHrodo™ Red zymosan (Thermo Fisher Scientific, P35364). Excess zymosan was removed by washing cells 3 times with PBS. Cells were then fixed by with cold 4% paraformaldehyde for 10 min. Afterwards, nuclei were stained with DAPI (Sigma, 1.5 μg/mL) and washed 3 times with PBS. Hemocytes were bathed in 200 μL PBS and visualized under a Leica SP8 confocal microscope.

RNA interference (RNAi)

To clarify the functional relevance of CLIC2α in oyster hemocytes, CLIC2α gene was knocked down *in vivo* via dsRNA-mediated RNA interference. The primers used to synthesize dsRNA are as shown in Table S1. A CLIC2α cDNA fragment and a GFP cDNA fragment (negative control) were amplified with primer pairs with T7 promoter overhangs (Promega, RiboMAX™ Express RNAi System). PCR products thus resulted were used as templates to synthesize dsRNA according to the manufacturer's instructions. Ten oysters were randomly assigned into 2 groups and placed in 2 tanks: the treatment and control groups. Each oyster was injected with 50 μg dsRNA and three individuals from each group were chosen randomly for the collection of hemocytes. Phagocytosis rate, bacterial clearance rate and degree of phagosomal acidification were evaluated 3 days after dsRNA injection. The expression level of CLIC2α was then determined by RT-qPCR and Western blot.

Total RNA extraction and quantitative real-time PCR analysis

Hemocytes were collected as above and total RNA was isolated with TRIzol Reagent (Invitrogen, 15596-026) according to the manufacturer's protocol. The RNA quality and quantity were detected using NanoDrop 2000C (Thermo Fisher Scientific, USA). Extracted RNA was converted to cDNA with the PrimerScript™ first strand cDNA synthesis kit (TAKARA, RR047A), then subjected to quantification analysis by using the 2×RealStar Green Power mixture (GenStar, A311) and LightCycler® 480 II (Roche, Switzerland) according to manufacturer's protocol. Primers were designed with Primer Premier (v5.0), and their sequences are as listed in Table S1. All experiments were performed in triplicates by using GAPDH mRNA as an internal control. Analysis of the dissociation curve of the amplification products was constructed to confirm specificity at the end of each PCR. Relative gene expression was calculated by using the $2^{-\Delta\Delta Ct}$ method. Data was represented by using SPSS10.0 statistical software, and significance between two groups was determined by Student's *t*-test.

Determination of phagosomal-lysosomal fusion

Hemocytes were cultured in 35-mm diameter glass-bottom dishes (NEST, 801001) and then incubated with 100 μM IAA-94 and the same dose of DMSO (solvent) for 30 min. Cells were then washed 3 times with PBS and further incubated with AlexaFluor-488-conjugated zymosan for an additional 20 min. Excess zymosan is removed by washing with PBS for 3 times. As per the manufacturer's protocol, cells were incubated with Lyso-Tracker Red (50 nM, Beyotime, C1046), a lysosomal red fluorescent probe for 30 min at room temperature in dark.

Subsequently, cells were fixed with cold 4% paraformaldehyde and their nuclei were stained with DAPI (Sigma, 1.5 μg/mL) as mentioned above. Imaging data were collected by using a Leica SP8 confocal microscope. Upon fusion, phagosomes (intracellular zymosan, green) co-localizing with lysosomes (red) appeared yellow.

Determination of HOCl levels *in vivo*

Hypochlorous acid (HOCl) levels in the samples were measured by a colorimetric MPO activity assay kit (Sigma, MAK068) according to the manufacturer's instructions. MPO (myeloperoxidase) catalyzes the formation of HOCl, which reacts with endogenous taurine to form taurine chloroamine. Taurine chloroamine reacts with the chromophore TNB, resulting in the formation of a colorless product, DTNB. Yields of this reaction can be approximately defined as the amount of taurine chloramine consuming the chromophore TNB. In brief, hemocytes after *in vivo* bacterial challenge were collected from oysters and homogenized in 4 equivalent volumes of MPO assay buffer. Among them, we used flow cytometry to ensure consistency of the number of sampled hemocytes. Then, hemocytes were centrifuged at 13,000×g for 10 min at 4°C to remove insoluble materials. Five microliters of the samples from supernatant (MPO substrate) were loaded into a 96-well microplate to incubate with MPO assay buffer at room temperature for 1 h, shielded from light. Then, 2 μL of a stop mix was added to the wells, followed by incubation for 10 min. Next, 50 μL of the TNB reagent/standard was added to the samples which were left to stand for an additional 10 min. Subsequently, absorbance at 412nm (A_{412}) was detected by means of an EnSightTM multimode plate reader (PerkinElmer, USA). Numerical data were obtained by comparing absorbance measurements against the standard curve.

ORF cloning and bioinformatics analysis

Partial cDNA sequence of *C. gigas* was BLAST searched in the oyster genome library (http://blast.ncbi.nlm.nih.gov/Blast.cgi). Based on identified sequences, the ORF of the CLIC2α genes were obtained by polymerase chain reaction (PCR), according to the manufacturer's instructions (GenStar, A012-01). Deduced amino acid sequences were obtained with the aid of ORF Finder (http://www.ncbi.nlm.nih.gov/gorf/orfig.cgi). A phylogenetic tree was constructed based on amino acid sequences of known CLICs proteins by performing Bayesian phylogenetic analysis. Further, transmembrane results were predicted with MemBrain (http://www.csbio.sjtu.edu.cn/bioinf/MemBrain).

Antibody production

The two amino acid sequences 1-129 a.a. and 201-292 a.a. in the ORF protein of CLIC2α were selected for protein expression and purification as antigens in mixed immunization. White rabbits are used as experimental animals for immunization. Multiple injections of a total amount of 600 μg of antigens and complete Freund's adjuvant were injected subcutaneously on the back of the rabbit. Injection proceeded once every two weeks, followed by evaluation of serum titers after four injections. Pre-sera were used as a negative control for antibody titer testing.

Rabbits were bled after passing the test. Then, protein Affinity purification, HABP affinity purification, and antigen affinity purification were performed successively to purify desired sera. The resultant purified antibodies were validated in immunoblotting and stored for subsequent experiments.

Immunofluorescence preparation

For imaging, hemocytes were fixed with cold 4% paraformaldehyde for 10 min, rinsed 3 times in PBS and permeabilized with 0.05% Triton in PBS for 15 min at room temperature. Then, hemocytes were blocked with QuickBlock™ blocking buffer (Beyotime, P0252) for 60 min, incubated overnight with appropriate primary antibodies at 4°C. CLIC2α was stained with an affinity purified rabbit polyclonal antibody (Sinobiological). After washing off primary antibodies, specimens were incubated in a fluorochrome-conjugated secondary antibody (Cell Signaling Technology, #4414) diluted in antibody dilution buffer for 1 h at room temperature in dark. DAPI staining was done to counterstain nuclei (Sigma, 1.5 µg/mL), followed by washing in PBS for 3 times. The hemocyte samples thus prepared were then immersed in 200 µL PBS and visualized under a Leica SP8 confocal microscope.

Confocal microscopy

Variations in Cl^- levels were monitored by using the fluorescent dye *N*-(ethoxycarbonylmethyl)-6-methoxyquinolinium bromide (MQAE, 5 mM, Beyotime, S1082), whose fluorescence becomes quenched via collision with chloride in oyster hemocytes. Experimentally, cells were allowed to engulf red-emission zymosan and fluorescent *E. coli* for a 30-min period to ensure that the majority of the immunostimulants have entered the phagosomal compartment. Fluorescence micrographs were acquired by using a Leica SP8 confocal fluorescence laser scanning system (Leica Microsystems, Germany). Lasers were used at the following excitation/emission parameters: λ_{ex} 405 nm for MQAE (λ_{em} 460 nm), λ_{ex} 560 nm for pHrodo™ Red zymosan (λ_{em} 585 nm), λ_{ex} 405 nm for DAPI (λ_{em} 454 nm), λ_{ex} 577 nm for Lyso-Tracker Red (λ_{em} 590 nm) and λ_{ex} 555 nm for RFP (λ_{em} 584 nm). Series of optical sections were collected and processed by using Image-Pro Plus 6.0. For analysis of imaging data, the bright field and fluorescence channels of representative images were superimposed to confirm cell morphological features and phagocytosis events. Each image regions of interest (ROIs) was selected in Image Pro Plus, corresponding to individual phagocytosis events or whole cells subjected to a particular treatment. To avoid bias, all individuals in the recording optical field were processed for data analysis except for: (1) cells with marked morphological alterations; or (2) aggregating cells. Typically, up to 5-20% of such visually deviant cells may be excluded from analysis (Jiang et al., 2012).

Statistical analysis

Data processing and statistical analyses were performed by using GraphPad Prism (version 8.0.1). All statistical values were expressed as the mean ± S.D., with the number of experiments (*n*) in parentheses. Significance between groups was determined by Student's *t*-

test, while comparisons for more than two groups were done by one-way ANOVA followed by Tukey's *post hoc* test with SPSS (version 22.0). Asterisks indicate a significance difference of $p < 0.05$.

Supplemental References

Duperthuy, M., Schmitt, P., Garzon, E., Caro, A., Rosa, R.D., Le Roux, F., Lautredou-Audouy, N., Got, P., Romestand, B., de Lorgeril, J.*, et al.* (2011). Use of OmpU porins for attachment and invasion of Crassostrea gigas immune cells by the oyster pathogen Vibrio splendidus. Proc Natl Acad Sci U S A *108*, 2993-2998.

Guckian, J.C., Christensen, W.D., and Fine, D.P. (1978). Trypan blue inhibits complement-mediated phagocytosis by human polymorphonuclear leukocytes. J Immunol *120*, 1580-1586.

Ikeuchi, Y., Kogiso, H., Hosogi, S., Tanaka, S., Shimamoto, C., Inui, T., Nakahari, T., and Marunaka, Y. (2018). Measurement of [Cl(-)]i unaffected by the cell volume change using MQAE-based two-photon microscopy in airway ciliary cells of mice. J Physiol Sci *68*, 191-199.

Jiang, L., Salao, K., Li, H., Rybicka, J.M., Yates, R.M., Luo, X.W., Shi, X.X., Kuffner, T., Tsai, V.W., Husaini, Y.*, et al.* (2012). Intracellular chloride channel protein CLIC1 regulates macrophage function through modulation of phagosomal acidification. J Cell Sci *125*, 5479-5488.

Saleh, M., Mathison, J.C., Wolinski, M.K., Bensinger, S.J., Fitzgerald, P., Droin, N., Ulevitch, R.J., Green, D.R., and Nicholson, D.W. (2006). Enhanced bacterial clearance and sepsis resistance in caspase-12-deficient mice. Nature *440*, 1064-1068.

Wang, X.W., Zhao, X.F., and Wang, J.X. (2014). C-type lectin binds to beta-integrin to promote hemocytic phagocytosis in an invertebrate. J Biol Chem *289*, 2405-2414.

Zhang, Y.L., Chen, P.X., Guan, W.J., Guo, H.M., Qiu, Z.E., Xu, J.W., Luo, Y.L., Lan, C.F., Xu, J.B., Hao, Y.*, et al.* (2018). Increased intracellular Cl(-) concentration promotes ongoing inflammation in airway epithelium. Mucosal Immunol *11*, 1149-1157.
